# Retractorless interforniceal approach for microsurgical resection of a papillary tumor of the pineal region: operative video and technical nuances

**DOI:** 10.3171/2021.4.FOCVID2139

**Published:** 2021-07-01

**Authors:** James K. Liu, Neil Majmundar

**Affiliations:** Department of Neurological Surgery, Center for Skull Base and Pituitary Surgery, Rutgers University, New Jersey Medical School, Newark; Saint Barnabas Medical Center, RWJ Barnabas Health, Livingston, New Jersey

**Keywords:** pineal region tumor, papillary tumor of the pineal region, anterior interhemispheric transcallosal interforniceal approach, posterior third ventricle, retractorless

## Abstract

In this illustrative video, the authors demonstrate microsurgical resection of a papillary tumor of the pineal region using a retractorless interforniceal approach via the anterior interhemispheric transcallosal route. The tumor presented to the posterior third ventricle occluding the cerebral aqueduct, resulting in obstructive hydrocephalus. The retractorless interforniceal approach is performed in the lateral position with BICOL collagen spacers to keep the corridor open. Gross-total resection was achieved, and the patient was neurologically intact without needing a permanent shunt. The operative nuances and pearls of technique for safe microdissection and gentle handling of the retractorless interforniceal approach are demonstrated.

The video can be found here: https://stream.cadmore.media/r10.3171/2021.4.FOCVID2139.

## Transcript

This is Dr. James Liu, and I will be demonstrating the retractorless interforniceal approach via the anterior interhemispheric transcallosal route for microsurgical resection of a papillary tumor of the pineal region.

### 0:35 Patient History and Neurological Examination

The patient is a 69-year-old male, who presented with gait ataxia and bilateral leg weakness. Neurological exam was nonfocal.

### 0:44 Preoperative Imaging

CT scan showed a solid pineal region mass occupying the posterior third ventricle resulting in obstructive hydrocephalus. MRI scan demonstrated an enhancing pineal region mass that was hyperintense on T1 and T2/FLAIR images with some enhancement after gadolinium. The initial differential diagnosis included germinomatous and nongerminomatous germ cell tumors, pineocytoma, and pineoblastoma.^1,2^ An endoscopic third ventriculostomy to treat the hydrocephalus, with simultaneous tumor biopsy, was performed. CSF as well as serum markers for germ cell tumors were sent, which came back negative. The final pathology was a papillary tumor of the pineal region. We therefore proceeded with definitive surgical resection of the tumor.

### 1:33 Choice of Surgical Approach

The following surgical approaches were considered for this lesion: the supracerebellar infratentorial approach, the occipital transtentorial approach, and the posterior interhemispheric transcallosal approach.^3–6^ Because of the location in the posterior third ventricle, my preference is the anterior interhemispheric approach using the retractorless interforniceal corridor.^7,8^ This approach provides a direct midline trajectory to the posterior third ventricle using the interforniceal and intervenous corridor. Although retractors are shown opening the interforniceal corridor in this Rhoton dissection, we will demonstrate this approach using a retractorless interforniceal technique.

### 2:21 Lateral Position and Skin Incision

The patient was placed in the lateral position with the right side down to allow gravity-assisted access to the interhemispheric fissure without fixed retraction. A bicoronal linear skin incision was made centered around the coronal suture. Image guidance was used to plan the transcallosal trajectory to the posterior third ventricular tumor. After making burr holes directly over the superior sagittal sinus, we elevated a right frontal craniotomy that crossed the midline to allow gentle retraction of the superior sagittal sinus to improve access to the interhemispheric fissure.

### 2:55 Dural Opening and Interhemispheric Exposure

A C-shaped dural incision was made and reflected toward the superior sagittal sinus. Using gravity assistance, the interhemispheric fissure is carefully opened with gentle spreading with bipolar forceps. Careful dissection and splitting of the fissure revealed the pericallosal arteries and the corpus callosum.

### 3:17 Corpus Callosotomy and Interforniceal Dissection

A corpus callosotomy of approximately 2 to 2.5 cm in length is made using bipolar cautery and suction. Both lateral ventricles are exposed and the midline septum pellucidum is visualized. The leaflets of the septum pellucidum are carefully separated using gentle spreading with a fine microbayonetted forceps to safely develop the interforniceal corridor. Dissection is aimed more posteriorly since the interforniceal distance is greater for safe entry. A BICOL collagen sponge is used as a space holder to keep the corridor open.

We now separate the leaflets of the tela choroidea, which forms the velum interpositum, which houses the internal cerebral veins. Care is taken to avoid injury to the veins and the medial posterior choroidal arteries.

### 4:10 Tumor Removal

The tumor is nicely exposed and visualized in the posterior third ventricle. We initially devascularize the tumor, then begin internal debulking with suction aspiration. You will notice that the tumor is soft, suckable, and very friable. The tumor is carefully delivered through the interforniceal corridor in a piecemeal fashion. The posterior aspect of the tumor is generally more adherent to the arachnoid membranes of the pineal recess and the tegmentum of the midbrain. Gentle dissection is used to separate the tumor from the floor of the third ventricle. Several tumor feeders from the branches of the medial posterior choroidal arteries are cauterized and sharply divided to further devascularize the tumor. Notice how we have maximized the interforniceal working corridor without the use of fixed retractors. This is largely due to the gravity assistance in the lateral position and the use of BICOL sponges. This meticulous “no-touch” technique avoids risk of interforniceal injury.

### 5:22 Final Inspection and Wound Closure

Final inspection shows a gross-total resection of the tumor and intact fornices bilaterally. We can now see the cerebral aqueduct, posterior commissure, and pineal recess. Hemostasis is achieved and each lateral ventricle is inspected for any hematoma. The third ventricle is clear of any hematoma upon a second inspection. The CSF spaces are recharged with saline and an external ventricular drain is placed under direct vision. Standard multilayered wound closure is performed.

### 6:06 Postoperative Imaging and Hospital Course

Postoperative MRI showed no evidence of residual tumor with resolution of hydrocephalus. Postoperatively, the patient was neurologically intact without any memory deficits. The ventricular drain was removed after a 24-hour clamp trial, and the patient was discharged home without needing a shunt.

### 6:26 Conclusion

In summary, the anterior interhemispheric transcallosal interforniceal approach is a useful strategy to surgically remove papillary tumors of the pineal region situated in the posterior third ventricle. This can be performed meticulously and safely in the lateral position using a retractorless interforniceal technique.

## Author Contributions

Primary surgeon: Liu. Assistant surgeon: Majmundar. Editing and drafting the video and abstract: both authors. Critically revising the work: both authors. Reviewed submitted version of the work: both authors. Approved the final version of the work on behalf of both authors: Liu. Supervision: Liu.

## References

[1] Yamaki VN Solla DJF Ribeiro RR Papillary tumor of the pineal region: systematic review and analysis of prognostic factors Neurosurgery 2019 85 3 E420 E429 30989225 10.1093/neuros/nyz062

[2] Fang AS Meyers SP Magnetic resonance imaging of pineal region tumours Insights Imaging 2013 4 3 369 382 23640020 10.1007/s13244-013-0248-6 PMC3675249

[3] Choque-Velasquez J Resendiz-Nieves J Jahromi BR Midline and paramedian supracerebellar infratentorial approach to the pineal region: a comparative clinical study in 112 patients World Neurosurg 2020 137 e194 e207 32001412 10.1016/j.wneu.2020.01.137

[4] Tanikawa M Yamada H Sakata T Exclusive endoscopic occipital transtentorial approach for pineal region tumors World Neurosurg 2019 131 167 173 31421299 10.1016/j.wneu.2019.08.038

[5] Liu JK Endoscopic-assisted interhemispheric parieto-occipital transtentorial approach for microsurgical resection of a pineal region tumor: operative video and technical nuances Neurosurg Focus 2016 40 Video Suppl 1 2016.1,15450 10.3171/2016.1.FocusVid.15450 26722692

[6] Patel PG Cohen-Gadol AA Mercier P The posterior transcallosal approach to the pineal region and posterior third ventricle: intervenous and paravenous variants Oper Neurosurg (Hagerstown) 2017 13 1 77 88 28931256 10.1227/NEU.0000000000001268

[7] Yağmurlu K Zaidi HA Kalani MYS Anterior interhemispheric transsplenial approach to pineal region tumors: anatomical study and illustrative case J Neurosurg 2018 128 1 182 192 28084911 10.3171/2016.9.JNS16279

[8] Siwanuwatn R Deshmukh P Feiz-Erfan I Microsurgical anatomy of the transcallosal anterior interforniceal approach to the third ventricle Neurosurgery 2005 56 2)(suppl 390 396 15794835 10.1227/01.neu.0000156842.84682.01

